# Technological Doping in Sport: Performance Enhancement, Health, Ethics, and Regulatory Governance: A Narrative Synthesis

**DOI:** 10.3390/bioengineering13030257

**Published:** 2026-02-24

**Authors:** Dan Iulian Alexe, Prashant Kumar Choudhary, Suchishrava Choudhary, Sohom Saha, Bindiya Rawat, Dragoș Ioan Tohănean, Ecaterina Lungu, Cristina Ioana Alexe

**Affiliations:** 1 Department of Physical and Occupational Therapy, “Vasile Alecsandri” University of Bacau, 600115 Bacău, Romania; alexedaniulian@ub.ro; 2 Department of Physical Education Pedagogy, Lakshmibai National Institute of Physical Education, Gwalior 474002, Madhya Pradesh, India; prashantlnipe2014@gmail.com (P.K.C.); suchishrava05@gmail.com (S.C.); 3 Department of Sports Psychology, Lakshmibai National Institute of Physical Education, Gwalior 474002, Madhya Pradesh, India; sohomsaha77@gmail.com; 4 Department of Liberal Arts and Social Sciences, Manipal University Jaipur, Jaipur 303007, Rajasthan, India; 5 Faculty of Physical Education and Mountain Sports, Transilvania University of Brașov, 500036 Brasov, Romania; 6 Department of Preschool Pedagogy, Physical Education and Dance, “Ion Creangă” State Pedagogical University of Chișinău, Str. Ion Creangă, Nr. 1, MD-2069 Chișinău, Moldova; lungu.ecaterina@upsc.md; 7 Department of Life Sciences, “Dunarea de Jos” University of Galati, Str. Domnească, Nr. 47, 800008 Galați, Romania; 8 Department of Physical Education and Sports Performance, “Vasile Alecsandri” University of Bacău, 600115 Bacău, Romania; alexe.cristina@ub.ro

**Keywords:** technological doping, sport technology, performance enhancement, anti-doping systems, wearable technologies, sports ethics, regulatory governance

## Abstract

Background: Technological innovation increasingly shapes modern sport, influencing performance, athlete safety, and regulatory governance. While new technologies enhance training and monitoring, they also raise concerns regarding fairness, health protection, and ethical legitimacy, commonly described as technological doping. The fragmented nature of the literature in this field requires integrative synthesis. Methods: A structured narrative synthesis was conducted using systematic searches and predefined eligibility criteria to identify studies addressing performance technologies, digital monitoring and detection systems, healthcare compliance, and governance and ethical frameworks. Twenty-four studies spanning empirical, policy, and conceptual domains were included. Results: Mechanical technologies, particularly advanced carbon-plate footwear, were associated with approximately 1–3% faster marathon performances and measurable alterations in lower-limb kinematics and kinetics under fatigue, while running-specific prostheses demonstrated performance-relevant differences in stiffness and energy return properties. Wearable monitoring systems supported training optimization but raised concerns related to surveillance and athlete autonomy. Artificial intelligence-based medication screening tools demonstrated high operational performance, with reported recognition accuracy ranging from approximately 92% to 98%, sensitivity approaching 1.00, and strong specificity for identifying prohibited substances from prescription images. Healthcare studies identified persistent knowledge gaps, medication risks, and the importance of pharmacists and education programs. Governance analyses revealed disparities in laboratory capacity and regulatory ambiguity when addressing emerging technologies, while ethical scholarship questioned the boundaries of legitimate enhancement. Conclusions: Technological doping reflects an interconnected performance–health–governance challenge rather than an isolated equipment issue. The synthesis demonstrates that technological doping is driven by measurable performance gains, digitally mediated compliance systems, and uneven regulatory capacity, indicating that future governance must shift from reactive equipment bans toward integrated, evidence-based oversight of biomechanical, digital, and healthcare technologies.

## 1. Introduction

Modern sport is increasingly shaped by advanced technologies that influence training, performance evaluation, and competitive outcomes beyond traditional physiological and psychological determinants [1,2]. In the context of this review, technological doping is operationally defined as the use or integration of sport-related technologies that confer a measurable or perceived performance advantage beyond the athlete’s physiological capacity, while simultaneously raising concerns regarding fairness, health protection, or regulatory legitimacy. This definition encompasses mechanical devices, digital monitoring systems, biomedical enhancement concepts, and technology-enabled compliance infrastructures, distinguishing technological doping from both conventional sport equipment innovation and pharmacological doping. By adopting this operational definition, the present synthesis focuses specifically on technologies that challenge existing boundaries between acceptable innovation and unfair advantage within competitive sport.

Wearable systems and artificial intelligence (AI) now support continuous monitoring, performance optimization, and injury prevention, transforming how athletes train and compete [3], while digital innovation also reshapes sport governance and commercialization structures [4]. These developments are increasingly embedded within broader processes of sport digitalization, including datafication, platform-based service models, and AI-enabled decision-support systems that extend beyond performance enhancement alone. Although technological progress expands performance potential, it simultaneously generates ethical, medical, and regulatory concerns, commonly described under the evolving concept of technological doping [1,5]. Unlike pharmacological doping, technological doping involves blurred boundaries between legitimate innovation and unfair advantage, creating persistent tension between progress, fairness, and athlete protection. Mechanical technologies represent some of the most visible sources of controversy within this broader ecosystem. Advanced carbon-plate footwear has been associated with faster running performances in elite competition [6,7], supported by biomechanical evidence indicating altered lower-limb mechanics under fatigue [8]. These developments have prompted regulatory debates regarding competitive equity and acceptable technological thresholds [9]. Similar concerns exist in para-sport, where running-specific prostheses demonstrate mechanical characteristics related to stiffness and energy return that can influence performance outcomes [10,11], and engineering improvements continue to enhance durability and mechanical efficiency [12,13,14]. While assistive devices are intended to restore functional capacity, their optimization for elite competition raises complex classification, governance, and fairness challenges that extend beyond equipment regulation and into the wider technological sport ecosystem [1].

This ecosystem-level perspective aligns with emerging frameworks such as Sports Industry 5.0, which conceptualize contemporary sport as a convergence of advanced digital technologies, sustainability imperatives, and human-centric values [4]. Within this paradigm, artificial intelligence, automation, and pervasive data systems coexist with heightened expectations regarding ethical legitimacy, inclusion, athlete well-being, and governance accountability [5]. Technological doping thus emerges not as a deviation from this transformation, but as a critical stress point within it, exposing tensions between innovation-driven performance gains and the normative foundations of fair and health-oriented sport [1,15]. Embedding technological doping within the sports Industry 5.0 transition enables a more comprehensive understanding of how digital infrastructures, regulatory logics, and ethical expectations interact across performance, health, and governance domains [4,16,17].

Healthcare systems play a critical role in technological doping governance. High rates of prescription medication use among athletes increase the risk of inadvertent violations [18], while knowledge gaps persist among athletes and medical professionals regarding anti-doping compliance [19,20]. Trust in doping control systems influences athlete cooperation [21], and pharmacists increasingly contribute to medication safety and education [22,23]. Preventive education programs have demonstrated reductions in doping risk behaviors [24], while public health evidence highlights broader societal implications of inadequate regulation [25]. Recent AI-based optical character recognition systems further support medication screening and compliance monitoring [2,26], illustrating how technology contributes both to performance enhancement and regulatory protection.

Despite extensive research across engineering, medicine, ethics, and policy, evidence remains fragmented across disciplinary domains, limiting regulators’ ability to align biomechanical evidence, healthcare risk management, digital monitoring, and ethical evaluation within coherent policy frameworks. No integrated narrative synthesis has comprehensively connected performance mechanisms, healthcare implications, regulatory capacity, and ethical considerations within a unified framework of technological doping. Therefore, this narrative synthesis aims to integrate empirical, conceptual, and policy-based evidence to examine how emerging technologies influence athletic performance, athlete safety, regulatory integrity, and ethical legitimacy while identifying future research priorities to safeguard fairness and health in technologically mediated sport.

## 2. Materials and Methods

### 2.1. Search Strategy and Selection Process

This narrative synthesis was conducted using a structured and transparent literature search strategy to ensure comprehensive identification of relevant evidence addressing technological doping in sport across performance enhancement, biomechanical mechanisms, healthcare implications, regulatory governance, and ethical considerations, including applied artificial intelligence-based technologies supporting athlete safety and anti-doping compliance [2]. Although the review adopted a narrative synthesis approach rather than a quantitative meta-analysis, methodological rigor was maintained through systematic searching, predefined eligibility criteria, independent screening, and structured data extraction, consistent with best-practice recommendations for high-quality narrative evidence synthesis and transparent reporting standards [27,28].

Selected elements of systematic review methodology, including the use of the PRISMA 2020 reporting framework and PICOS-based eligibility logic, were intentionally adopted to enhance transparency and reproducibility in study identification and selection [29]. However, this review does not constitute a full systematic review, as it does not include quantitative meta-analysis, formal risk-of-bias assessment, or statistical certainty grading. These components were not applied due to the high heterogeneity of included evidence and the intentional inclusion of conceptual, ethical, legal, and policy-oriented literature alongside empirical biomechanical and technological studies. A narrative synthesis approach was therefore methodologically preferable, as it enables integrative interpretation across diverse disciplinary domains that cannot be meaningfully pooled statistically, consistent with established guidance for mixed-evidence narrative reviews [27,28,29]. Beyond thematic grouping, evidence synthesis was conducted through an integrative, cross-domain analytical process. Findings from each domain (mechanical performance technologies, digital and AI-based systems, healthcare and compliance interfaces, and governance and ethical considerations) were not interpreted in isolation but examined for conceptual linkages, reciprocal influences, and shared governance implications. This approach enabled the identification of cross-cutting patterns, such as how performance-enhancing technologies interact with regulatory capacity, healthcare vulnerabilities, and ethical legitimacy. The synthesis, therefore, emphasizes relational interpretation across domains rather than parallel thematic description, supporting an ecosystem-level understanding of technological doping in sport.

#### 2.1.1. Data Sources and Databases

Electronic literature searches were conducted across multiple international bibliographic databases to ensure comprehensive coverage of sport science, medicine, engineering, ethics, and policy-related literature relevant to technological doping in sport. The databases systematically searched included PubMed/MEDLINE (a core biomedical and life sciences database), Scopus, Web of Science Core Collection (major multidisciplinary citation indexes), Google Scholar (used selectively to identify supplementary peer-reviewed and early-access publications), the MDPI database, the Frontiers journal platform, and the CrossRef metadata search, in line with recommended systematic review search practices that emphasize breadth and cross-disciplinary retrieval [29]. In addition to electronic searching, targeted manual screening of reference lists from key review articles and highly cited publications was undertaken using a snowballing approach to capture potentially relevant studies not retrieved during the initial search process, a recognized strategy for increasing search sensitivity [30]. Regulatory and governance documents were cross-verified using official organizational sources where applicable to ensure accuracy and authenticity. The search strategy covered publications from approximately January 2000 to March 2025, reflecting the rapid evolution of technological innovation within modern sport. Only English-language publications with accessible full-text availability were considered eligible for inclusion [29]. To mitigate the risk of over-specific retrieval, footwear-related search terms were deliberately complemented by broader descriptors (e.g., “advanced footwear” and “performance-enhancing technology”), ensuring inclusion of diverse mechanical innovations beyond individual product categories.

#### 2.1.2. Search Terms and Boolean Strategy

A comprehensive keyword strategy was developed using a combination of controlled vocabulary terms and free-text keywords to maximize sensitivity while maintaining conceptual precision. Search strings were adapted slightly across databases to accommodate platform-specific indexing structures while preserving consistent thematic coverage. Three primary keyword clusters were employed: (i) technology and performance enhancement terms, including “technological doping,” “performance enhancing technology,” “sport technology,” “carbon plate shoes,” “advanced footwear,” “prosthetic running blades,” “wearable sensors,” “artificial intelligence in sport,” and “digital health in sport”; (ii) anti-doping and governance terms, including “anti-doping technology,” “doping detection systems,” “optical character recognition,” “AI drug recognition,” “anti-doping compliance,” “doping control laboratories,” “World Anti-Doping Agency,” “Court of Arbitration for Sport,” and “regulatory governance”; and (iii) ethics and biomedical enhancement terms, including “gene doping,” “human enhancement,” “ethical implications in sport,” “fairness in sport technology,” and “biomedical enhancement.” A representative Boolean search string applied in Scopus combined these clusters as follows: (“technological doping” OR “sport technology” OR “carbon plate footwear” OR prosthe* OR wearable* OR “artificial intelligence” OR OCR) AND (“performance enhancement” OR “anti-doping” OR compliance OR governance OR ethics OR regulation). Search strategies were iteratively refined through pilot testing to optimize the balance between sensitivity and specificity. Truncation symbols, proximity operators, and field-specific filters were applied where supported by individual databases to enhance retrieval precision.

#### 2.1.3. Identification and De-Duplication

The combined electronic and supplementary search strategy yielded a total of 198 records, including 186 records identified through database searching and 12 records obtained from registers and manual reference screening. All retrieved citations were exported into a reference management software for systematic organization and duplicate detection, consistent with recommended systematic review procedures that emphasize rigorous record management before screening [29]. Automated duplicate identification was performed initially, followed by manual verification to ensure accuracy, which is considered best practice to maximize deduplication efficiency and minimize reviewer burden in interdisciplinary systematic reviews [31]. A total of 38 duplicate records were removed. Subsequently, 22 records were flagged as ineligible by automated screening tools due to document type mismatch, incomplete metadata, or classification as non-academic sources, reflecting standardized record-triage procedures before formal screening [29,32]. An additional 18 records were removed for other reasons, including irretrievable abstracts, inaccessible full-text availability, or clear irrelevance to sport or technological contexts, in accordance with PRISMA reporting recommendations for documenting exclusions before screening [29]. Following this pre-screening process, 120 unique records remained and were advanced for formal title and abstract screening.

#### 2.1.4. Screening Process

Title and abstract screening were independently conducted by two reviewers using the predefined inclusion and exclusion criteria. During this stage, records were evaluated for relevance to competitive sport contexts, explicit engagement with technological systems influencing performance, detection, governance, or ethics, and the presence of analytical or empirical content rather than purely descriptive or journalistic reporting. Studies clearly unrelated to sport technology, anti-doping systems, biomedical enhancement, or healthcare applications were excluded, following procedures commonly implemented in contemporary systematic reviews in sport science that adhere to PRISMA guidance and structured screening workflows [33,34]. Following this screening stage, 74 records were excluded due to insufficient relevance, lack of technological focus, or inadequate methodological depth. The remaining 46 records were retained for full-text retrieval and eligibility assessment. Any discrepancies between reviewers were resolved through structured discussion to achieve consensus and ensure consistency of decision-making, consistent with recommended practices for systematic evidence synthesis in sport and exercise research [33].

#### 2.1.5. Full-Text Eligibility Assessment

**Figure 1:**
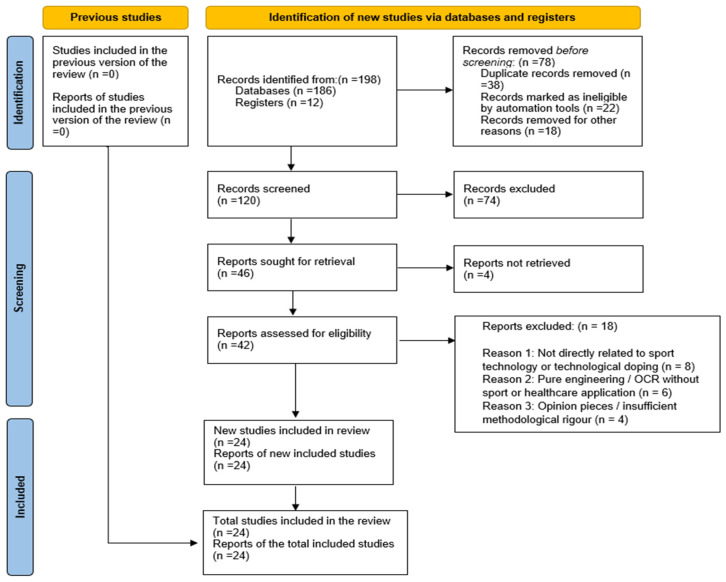
PRISMA 2020 flow diagram.

Full-text versions of all potentially eligible studies were obtained whenever possible. Of the 46 reports sought for retrieval, four could not be accessed due to unavailable publisher archives or restricted access limitations, resulting in 42 full-text articles assessed for eligibility. Full-text screening was conducted independently by both reviewers using a detailed eligibility framework based on PICOS logic and PRISMA guidelines, consistent with methodological standards commonly applied in sport-related systematic reviews [35,36]. Studies were excluded if they met any of the following criteria: (i) lack of direct relevance to sport technology or technological doping (n = 8), (ii) pure engineering or optical character recognition studies without sport or healthcare application (n = 6), or (iii) opinion-based publications or studies with insufficient methodological rigor (n = 4). Disagreements were resolved through reviewer discussion and consensus, in accordance with recommended practices for systematic evidence synthesis [36]. In total, 18 studies were excluded at this stage. Following this process, 24 studies met all inclusion criteria and were retained for narrative synthesis. The complete selection process is illustrated in the PRISMA 2020 flow diagram (Figure 1).

#### 2.1.6. Evidence Appraisal Strategy

A formal quantitative quality appraisal or risk-of-bias assessment was not conducted in this narrative synthesis due to the intentional inclusion of highly heterogeneous study designs, including experimental biomechanics, observational performance analyses, system validation studies, policy analyses, and conceptual ethical frameworks. Standardized appraisal tools are not equally applicable across such diverse methodological traditions and may produce misleading equivalence between empirical and normative evidence. Instead, an interpretive appraisal strategy was applied, whereby studies were categorized according to their primary contribution domain (mechanical performance, digital systems, healthcare and compliance, governance, or ethics), and findings were synthesized within these thematic categories without weighting evidence hierarchically. This approach minimized inappropriate comparisons across fundamentally different evidence types and allowed for the balanced integration of empirical results with policy and ethical scholarship, consistent with the recommendations of narrative synthesis methodology [27,28]. Although a formal standardized quality appraisal tool (e.g., risk-of-bias scales or evidence hierarchies) was not applied, methodological quality was considered at an interpretive level during synthesis. Studies were evaluated with attention to study design appropriateness, transparency of methods, relevance to competitive sport contexts, and plausibility of reported outcomes. Empirical studies were interpreted in light of their design limitations (e.g., observational versus experimental), while conceptual, ethical, and policy analyses were assessed based on analytical rigor and relevance to governance challenges. This pragmatic appraisal approach aligns with recommended practices for narrative syntheses incorporating heterogeneous evidence types while avoiding inappropriate equivalence across fundamentally different methodological traditions. Importantly, exclusion of non-peer-reviewed opinion editorials did not preclude inclusion of normative ethical scholarship, as peer-reviewed conceptual, philosophical, and policy analyses were explicitly retained to support ethical interpretation.

Although tools such as the Mixed Methods Appraisal Tool (MMAT) (2018 version) are valuable for reviews integrating qualitative and quantitative empirical studies, their application remains limited when synthesizing heterogeneous evidence that includes conceptual ethics, legal analysis, and policy evaluation. Applying a single standardized appraisal instrument across such epistemologically distinct evidence types risks producing misleading assessments of rigor. The present review, therefore, prioritized transparency of study design, funding context, and analytical plausibility during interpretation, enabling readers to critically appraise the strength and origin of evidence underpinning the synthesis.

#### 2.1.7. Protocol Registration and Synthesis Procedure

The review protocol was not registered in PROSPERO or a comparable registry, as registration is not mandatory for narrative syntheses, and the present review integrated empirical, policy, and ethical literature that falls outside typical biomedical systematic review frameworks. However, all eligibility criteria, databases, and screening procedures were defined a priori to reduce selection bias and enhance transparency. For evidence integration, a structured thematic synthesis approach was applied. Following a full-text eligibility assessment, included studies were grouped into four predefined analytical domains: mechanical performance technologies, digital and AI-based systems, healthcare and compliance interfaces, and governance and ethical considerations. Findings were extracted and compared within each thematic domain to identify convergent patterns, recurring challenges, and domain-specific implications, without quantitative pooling or hierarchical weighting. This approach enabled integrative interpretation across heterogeneous evidence types while maintaining conceptual coherence across disciplines.

## 3. Results

Across the 24 included studies summarized in Table 1 and Table 2, technological doping in sport emerged as a multidimensional phenomenon encompassing mechanical performance enhancement, digital monitoring and detection systems, healthcare compliance, and governance and ethical frameworks. Across thematic domains, mechanical performance findings were primarily supported by experimental and observational studies, digital and compliance systems by system development and validation studies, healthcare insights by surveys and epidemiological reviews, and governance and ethical perspectives by legal analyses and conceptual scholarship, reflecting domain-specific evidence traditions rather than uniform methodological hierarchies. Although presented by domain, these findings reveal substantial interdependence, with performance technologies influencing healthcare risk, digital compliance systems shaping governance capacity, and ethical considerations permeating all technological domains.

### 3.1. Mechanical Performance Technologies

Mechanical performance technologies were most prominently represented by advanced footwear and prosthetic systems. Observational analyses of elite and sub-elite marathon data reported that carbon-plate footwear was associated with approximately 1–3% faster race times compared with conventional footwear [6], while predictive modeling indicated an increased probability of achieving sub-two-hour marathon performances with advanced shoe technology [7]. Controlled biomechanical investigations further demonstrated that variations in carbon-fiber plate geometry altered lower-limb kinematics and kinetics under fatigue conditions, suggesting altered mechanical efficiency during running [8]. Policy-oriented analyses documented regulatory responses and ongoing fairness concerns surrounding footwear innovation in elite competition [9]. In para-sport contexts, experimental mechanical testing revealed significant differences in stiffness, hysteresis, and energy storage properties across running-specific prostheses [10]. Engineering evaluations confirmed improvements in durability and mechanical reliability of carbon-fiber prosthetic blades under repeated loading conditions [12]. Systematic synthesis of biomechanical studies further indicated that prosthetic stiffness and energy return characteristics were associated with variations in running performance outcomes [11].

### 3.2. Digital and AI-Based Monitoring and Detection Systems

Digital augmentation through wearable technologies was reported to facilitate physiological monitoring, workload tracking, and performance optimization across multiple sport contexts [13]. Studies described the use of sensor-based platforms for real-time feedback on movement, heart rate, and training load, enabling individualized training adjustments. However, reviews of wearable technology also reported concerns related to continuous data capture, including risks associated with surveillance, data ownership, and athlete autonomy [14]. Artificial intelligence based anti-doping technologies were represented by system development and validation studies using deep learning driven optical character recognition (OCR). These systems demonstrated high operational performance, with reported recognition accuracy ranging from approximately 92% to 98%, sensitivity approaching 1.00, and strong specificity for identifying banned substances from prescription images [2,26]. These platforms were designed to support scalable medication screening and compliance monitoring for athletes and support staff.

### 3.3. Healthcare, Medication Use, and Compliance Interfaces

This domain was included specifically to examine how healthcare interfaces function as technology-mediated decision-support systems within technological doping governance, rather than as traditional behavioral anti-doping research. From a healthcare perspective, narrative and epidemiological studies reported high prevalence of prescription medication use among athletes, increasing the risk of inadvertent anti-doping violations [18]. Survey-based investigations identified persistent knowledge gaps and misconceptions among athletes regarding prohibited substances and anti-doping regulations [19], as well as variable confidence among medical practitioners in providing accurate anti-doping guidance [20]. Qualitative investigations further indicated that athletes’ trust in doping control systems influenced compliance behaviors and perceived legitimacy of enforcement mechanisms [21]. Pharmacists were identified as key advisory resources for medication guidance in athletic populations, with surveys and professional frameworks highlighting their expanding role in anti-doping education and pharmaceutical care [22,23]. Preventive intervention studies integrating education and structured training programs reported reductions in doping-related risk behaviors among participants [24]. At the population level, international public health reviews documented widespread exposure to doping behaviors across athletic and non-athletic populations [25].

### 3.4. Governance Structures and Ethical Considerations

At the governance level, laboratory capability assessments revealed variability in technological readiness, analytical capacity, and infrastructure across anti-doping laboratories internationally [17]. Legal analyses of arbitration cases identified interpretative ambiguities within the World Anti-Doping Code when applied to technologically complex or novel enhancement contexts [16]. Broader classifications of sport technology controversies also emphasized regulatory uncertainty when technological innovation outpaces policy development [1]. This governance complexity is further contextualized within the emerging Sports Industry 5.0 paradigm, which emphasizes the integration of advanced technologies, human-centric values, and sustainability in sport ecosystems, reinforcing the need for adaptive and ethically grounded regulatory frameworks [37]. Ethical and biomedical scholarship highlighted unresolved tensions between enhancement and legitimacy in technologically augmented sport. Conceptual analyses addressed the moral boundaries of gene-based enhancement and potential long-term health implications [15], while philosophical critiques questioned traditional definitions of fairness and human performance under conditions of technological augmentation [5]. Although empirical sport-specific evidence on gene doping remains limited, contemporary ethical debates increasingly reference genome-editing technologies such as CRISPR/Cas9 as plausible future enhancement modalities, reinforcing the relevance of earlier conceptual analyses to emerging biomedical realities.

Table 2 summarizes the principal characteristics of the 24 studies included in the narrative synthesis, detailing the type of technology examined, the sport or application context, study design, sample or data source, key outcomes, and relevance to technological doping. The included studies encompass a wide range of methodological approaches, including observational analyses, experimental and biomechanical investigations, system development and validation studies, integrative and narrative reviews, policy analyses, and conceptual ethical frameworks.

Technologies represented span performance-enhancing mechanical systems (advanced footwear and prosthetic devices), wearable and digital monitoring platforms, artificial intelligence-based anti-doping tools, biomedical enhancement perspectives, and governance and regulatory infrastructures. The diversity of study designs and outcome measures reflects the interdisciplinary nature of technological doping research, supporting a comprehensive synthesis of performance, health, ethical, and regulatory implications across various sport contexts.

## 4. Discussion

This narrative synthesis indicates that technological doping in sport should not be understood as an isolated issue linked only to specific equipment but rather as a complex and evolving ecosystem in which mechanical innovation, digital intelligence, biomedical ethics, healthcare systems, and regulatory governance interact to shape competitive integrity and athlete welfare. The convergence of these domains suggests that performance enhancement and regulatory protection are increasingly intertwined, requiring coordinated and adaptive oversight mechanisms rather than fragmented policy responses. Rather than restating domain-specific findings, the following discussion critically evaluates how these results interact to expose structural limitations in current governance and regulatory models.

Technological doping in sport increasingly reflects the dynamics of digitally transformed sport ecosystems rather than isolated technological interventions. As sport organizations adopt integrated data platforms, wearable monitoring systems, AI-based analytics, and connected infrastructures, the governance of performance enhancement becomes embedded within complex socio-technical systems. These digital infrastructures reshape power relations by expanding surveillance capacities, centralizing data ownership, and enabling algorithmic decision-making that influences training, selection, and regulatory compliance. While such systems enhance performance optimization and risk management, they also constrain athlete autonomy and raise concerns regarding consent, transparency, and equitable access.

Within the context of Sports Industry 5.0, these developments highlight the dual role of technology as both an enabler of performance and a mechanism of governance. AI-driven compliance tools, such as medication screening and monitoring platforms, demonstrate how digital systems can strengthen regulatory capacity and reduce inadvertent anti-doping violations. However, the same infrastructures may intensify monitoring practices that blur boundaries between protection and control. Future governance models must therefore integrate biomechanical evidence, digital compliance tools, data governance principles, and ethical oversight within coherent regulatory ecosystems, rather than addressing each domain in isolation.

From a research perspective, this transformation underscores the need for cross-disciplinary collaboration between sport technologists, data governance scholars, ethicists, and sport management researchers. Key research priorities include examining how human-centric design principles can mitigate surveillance risks associated with wearable technologies, how algorithmic transparency influences trust in digital anti-doping systems, and how Industry 5.0-oriented governance frameworks can balance innovation with athlete well-being and fairness. Addressing technological doping as an ecosystem-level phenomenon thus requires governance strategies capable of evolving alongside digitally mediated sport environments.

Mechanical technologies continue to represent the most visible sources of performance-related controversy, particularly in endurance sports and para-sport disciplines. Rather than constituting incremental equipment upgrades, recent innovations have altered fundamental biomechanical and energetic characteristics of movement, raising persistent concerns regarding equitable competition and standardization of technological thresholds. The para-sport context further complicates regulatory classification, as assistive devices simultaneously function as mobility restorers and performance determinants, making clear separation between rehabilitation and enhancement difficult. These dynamics highlight the need for sport-specific, evidence-informed classification systems that evolve alongside engineering innovation rather than relying on static equipment regulations.

Digital technologies expand the technological footprint of sport beyond physical devices, introducing continuous monitoring, performance analytics, and decision-support tools into everyday training and competition environments. While these systems offer meaningful opportunities for individualized training optimization and injury prevention, they also introduce ethical challenges related to surveillance, data ownership, informed consent, and power asymmetries between athletes and organizations. The integration of wearable technologies therefore raises governance questions that extend beyond competitive fairness to include labor rights, data protection, and athlete autonomy, suggesting that ethical frameworks must evolve in parallel with technical capability. In jurisdictions governed by data protection frameworks such as the General Data Protection Regulation (GDPR), continuous athlete monitoring raises unresolved questions regarding lawful data processing, employer–employee power asymmetries, and ownership of biometric performance data.

Importantly, technology also plays a protective and preventive role within anti-doping systems. Artificial intelligence-driven medication screening and digital compliance platforms illustrate how technological innovation can strengthen regulatory capacity and reduce inadvertent violations. These tools address longstanding healthcare vulnerabilities, including inconsistent access to expert advice, limited anti-doping literacy, and high medication exposure among athletes. However, technological solutions alone are insufficient if not embedded within trusted institutional systems. Athlete confidence in testing procedures and regulatory fairness remains central to compliance behavior, underscoring the importance of transparency, education, and consistent enforcement practices alongside digital tools.

From a governance perspective, disparities in laboratory infrastructure and analytical capacity reveal structural inequalities that may affect detection reliability and procedural justice across jurisdictions. Although the reviewed studies did not provide systematic regional comparisons, available evidence suggests that disparities in laboratory infrastructure disproportionately affect low- and middle-income regions, potentially undermining global procedural equity in anti-doping enforcement. Legal ambiguities in the application of anti-doping codes to technologically mediated cases further illustrate how regulatory frameworks frequently lag behind innovation. Ethical debates surrounding biomedical enhancement, including gene-based interventions, challenge traditional assumptions about merit, effort, and human limitation, indicating that future regulatory decisions must address not only detection feasibility but also normative definitions of legitimate performance.

Collectively, these findings suggest that technological doping cannot be effectively managed through isolated equipment bans or reactive policy updates. Instead, adaptive governance models are required that integrate biomechanical evidence, digital compliance systems, healthcare safeguards, legal clarity, and ethical deliberation into unified regulatory strategies. One potential model is a “Technological Passport,” analogous to the Athlete Biological Passport, in which approved performance technologies are longitudinally monitored for biomechanical impact, usage patterns, and regulatory compliance, enabling proactive oversight rather than retrospective prohibition.

Such integration would allow governing bodies to respond more dynamically to emerging technologies while maintaining fairness, safety, and public trust. Several practical implications arise from this synthesis. Regulatory authorities should prioritize proactive technology assessment frameworks rather than retrospective rule modification. Digital monitoring and medication screening systems should be deployed with strict safeguards for privacy, data security, and informed consent. Healthcare professionals and pharmacists require standardized digital decision-support tools and continuing education to prevent inadvertent violations. Laboratory infrastructure must be continuously updated to ensure equitable detection capacity, and legal standards must evolve to address novel enhancement modalities. Finally, ethical evaluation should remain central to policy development, ensuring that innovation does not erode the foundational values of sport.

### 4.1. Practical Implications

From a policy perspective, governing bodies should adopt proactive technology assessment frameworks that evaluate performance effects, accessibility, and safety before widespread competitive adoption, rather than relying on retrospective rule modifications. Regulatory agencies should also prioritize harmonization of laboratory infrastructure and detection capacity to reduce procedural inequities across regions.

In clinical and healthcare practice, standardized digital decision-support systems for medication screening should be integrated into athlete healthcare services, with pharmacists and sports medicine professionals receiving continuous education on evolving prohibited substance lists and therapeutic use regulations. Such integration may substantially reduce inadvertent violations and improve athlete safety.

For coaches and performance practitioners, wearable and monitoring technologies should be applied within ethically transparent frameworks that ensure informed consent, data protection, and athlete agency. Training programs should balance data-driven optimization with psychological well-being and autonomy, avoiding excessive surveillance that may undermine trust and motivation.

### 4.2. Limitations

Several limitations of the present narrative synthesis should be acknowledged. First, the included studies demonstrated substantial methodological heterogeneity, limiting direct comparability across performance, biomedical, and policy domains. Second, the absence of a formal risk-of-bias assessment restricts the ability to evaluate the internal validity of individual empirical findings. Third, reliance on English-language publications may have excluded relevant regulatory and technological evidence from non-English jurisdictions. Fourth, longitudinal health consequences of technologically enhanced performance remain insufficiently studied, limiting conclusions regarding long-term athlete welfare. Finally, regulatory capacity and ethical governance structures evolve rapidly, meaning that some policy interpretations may become outdated as technology and institutional frameworks continue to develop.

## 5. Conclusions

This narrative synthesis highlights that technological doping in sport represents a multifaceted challenge extending beyond isolated performance devices to encompass mechanical innovation, digital intelligence, biomedical ethics, healthcare delivery, and regulatory governance. Evidence demonstrates that advanced footwear and prosthetic technologies can produce measurable performance advantages through altered biomechanics and mechanical efficiency, raising persistent concerns regarding fairness and competitive equity. Concurrently, wearable systems and artificial intelligence-driven tools offer powerful opportunities for performance optimization, medication safety, and anti-doping compliance, reinforcing the dual role of technology as both an enabler and regulator of athletic performance. Healthcare systems, particularly pharmacists and sports medicine practitioners, emerge as critical interfaces for preventing inadvertent violations and safeguarding athlete health, while education and trust in doping control systems influence long-term compliance behaviors. Regulatory analyses reveal that institutional capacity and legal frameworks often lag behind technological advancement, underscoring the need for adaptive governance mechanisms informed by empirical evidence and ethical reflection. Collectively, these findings emphasize that effective management of technological doping requires integrated strategies that align scientific validation, healthcare protection, digital innovation, and transparent regulation. Future efforts should prioritize interdisciplinary collaboration, continuous technological surveillance, and harmonized policy development to ensure that innovation enhances performance safely while preserving the integrity and social legitimacy of sport.

Taken together, the central contribution of this review is the demonstration that technological doping is not a problem of isolated devices or rule violations, but a systemic governance challenge emerging from digitally mediated sport ecosystems. The interaction of engineering innovation, data-driven monitoring, healthcare interfaces, and evolving regulatory frameworks reflects broader transformations associated with the transition toward Sports Industry 5.0. Understanding technological doping within this ecosystem perspective shifts attention from single-technology bans toward integrated governance models that align scientific validation, digital infrastructure, ethical oversight, and athlete-centered design. Such an approach is essential to ensure that technological innovation enhances performance responsibly while preserving fairness, athlete welfare, and the social legitimacy of sport in an increasingly digital era.

## Figures and Tables

**Table 1:** Inclusion and Exclusion Criteria for the Narrative Synthesis.

Domain (PICOS Logic)	Inclusion Criteria	Exclusion Criteria
Population/Context	Competitive athletes (elite, sub-elite, para-athletes, youth). Sport governance bodies, anti-doping agencies, laboratories, healthcare professionals, pharmacists. Sport technology environments influencing athlete performance, safety, fairness, or compliance.	Non-sport populations. Recreational fitness without competitive relevance. Clinical populations unrelated to sport performance or anti-doping.
Technology Exposure	Performance-enhancing technologies (carbon-plate footwear, prosthetic blades, wearable sensors, smart equipment). Digital and AI technologies supporting doping detection, compliance, or monitoring (OCR systems, laboratory analytics). Biomedical enhancement technologies (gene enhancement concepts). Regulatory and governance technologies influencing enforcement and compliance systems.	Non-sport engineering technologies. General OCR, AI, or engineering studies without a sport or anti-doping application. Consumer fitness gadgets without performance or governance implications.
Comparator/Perspective	Empirical comparisons (technology vs. non-technology). Modeling or simulation comparisons. Policy, ethical, and governance evaluations. Pre–post or cross-sectional evaluations.	Studies lacking analytical comparison or interpretive framework. Pure descriptive news articles or opinion editorials.
Outcomes of Interest	Performance enhancement (speed, efficiency, mechanical advantage, marginal gains). Biomechanical and physiological mechanisms. Health, safety, and injury implications. Ethical fairness and integrity implications. Regulatory effectiveness and governance challenges. Technology-supported compliance and detection accuracy.	Outcomes unrelated to performance, health, governance, or technological influence. Pure sociological or historical narratives without technology linkage.
Study Design	Observational studies. Experimental and biomechanical studies. System development and validation studies. Systematic, integrative, and narrative reviews. Policy analyses and conceptual ethics papers.	Case reports without analytical depth. Conference abstracts only. Non-peer-reviewed blog posts or media commentary.
Publication Characteristics	Peer-reviewed journal articles and high-quality academic books. English language. Full-text available. Publication period approximately 2000 2025.	Non-English publications. Gray literature without methodological transparency. Duplicate publications.
Relevance Threshold	Direct contribution to understanding technological doping, performance enhancement technologies, or technology-driven governance in sport.	General doping physiology, psychology, or sociology without technological focus.

**Table 2:** Characteristics of Included Studies on Technological Doping in Sport (n = 24).

Author (Year)	Technology Category	Sport/Context	Study Design	Sample/Data	Key Outcomes	Relevance to Technological Doping
Guinness et al. (2020) [6]	Carbon-plate footwear	Marathon running	Observational performance analysis	Elite and sub-elite marathon results (large dataset)	Vaporfly is associated with ~1 3% faster marathon times	Direct evidence of technology-driven performance enhancement
Lee et al. (2023) [2]	AI-based OCR system	Anti-doping compliance in athletes	System development and validation study	886 prescription/drug images; 336 banned substances database	Character recognition accuracy 98.3%; system accuracy 0.95; sensitivity 1.00; specificity 0.93	Demonstrates how advanced digital technology supports doping prevention, athlete safety, and regulatory compliance
Arderiu & De Fondeville (2022) [7]	Advanced footwear	Elite marathon modeling	Statistical modeling (preprint)	World-class performance datasets	Advanced footwear increases probability of sub-2-hour marathon	Quantifies technological advantage
Dyer (2020) [9]	Footwear regulation	Policy analysis	Narrative policy review	Regulatory documents + case analysis	Highlights unfair advantage, regulatory responses	Ethical and governance implications
Xu et al. (2025) [8]	Carbon-fiber plate biomechanics	Running biomechanics	Controlled experimental study	12 trained runners	Carbon-fiber plate geometry significantly altered ankle and knee joint kinematics and joint moments under fatigue, indicating changes in mechanical efficiency during running.	Mechanistic basis of performance advantage
Dyer (2015) [1]	Sports technology	Multi-sport	Systematic review	56 studies	Identifies controversial sport technologies	Foundational classification of technological doping
Rahnama et al. (2024) [11]	Running prostheses	Para-athletics	Systematic review	Human biomechanical studies	Prosthetic stiffness and energy return affect performance	Assistive tech vs. unfair enhancement
Siddiqui et al. (2023) [12]	Prosthetic blade engineering	Prosthetic design	Mechanical testing	Carbon-fiber blade prototype	Improved reliability and mechanical durability	Performance durability implications
Beck et al. (2016) [10]	Running-specific prostheses	Para-running	Experimental mechanical testing	Multiple prosthetic models	Quantified stiffness, hysteresis, energy storage	Objective mechanical advantage analysis
Migliaccio et al. (2024) [13]	Wearable technology	Multi-sport	Integrative review	Sensor-based systems	Wearables enhance marginal performance gains	Data-driven augmentation
Seçkin et al. (2023) [14]	Wearable sensors	Sports monitoring	Narrative review	Multiple device types	Benefits and ethical risks of continuous monitoring	Technological surveillance concerns
Triviño (2011) [15]	Gene doping ethics	Biomedical enhancement	Conceptual analysis	Policy and ethics literature	Ethical boundaries of genetic enhancement	Biomedical technological doping
Miah (2006) [5]	Human enhancement	Sport ethics	Philosophical review	Theoretical framework	Challenges traditional fairness concepts	Conceptual grounding
Pitassi & Lacerda (2019) [17]	Anti-doping laboratory tech	Policy systems	Metrics development study	Global labs	Measures tech capacity of doping labs	Governance and detection infrastructure
Baron et al. (2007) [25]	Doping epidemiology	Public health	International review	Multiple countries	Spread of doping behaviors	Contextual health risk framing
Pavot (2022) [16]	Anti-doping regulation and legal interpretation	Elite international sport (CAS/WADA governance)	Legal and policy analysis	CAS arbitration cases (Kamila Valieva case)	Identified regulatory gaps and interpretative ambiguity in the World Anti-Doping Code	Demonstrates regulatory vulnerability and governance challenges surrounding modern doping and technology
Park et al. (2022) [26]	AI-based OCR system	Anti-doping drug identification	System development study	Prescription and drug images (Korean language database)	OCR-based system achieved ~92% accuracy in classifying banned vs. acceptable substances	Early digital anti-doping technology supporting medication safety and compliance
Kim & Kim (2017) [19]	Athlete knowledge and compliance systems	National-level athletes (Korea)	Cross-sectional survey	Competitive athletes	Limited knowledge and misconceptions regarding doping regulations	Supports need for technological education and digital compliance tools
Overbye (2016) [21]	Doping control system trust	Elite athletes	Qualitative investigation	Interviews with elite athletes	Trust and perceived fairness influence compliance behavior	Highlights human technology interaction in doping control systems
Backhouse & McKenna (2011) [20]	Medical decision-support systems	Sports medicine practitioners	Narrative review	Healthcare professionals	Knowledge gaps in anti-doping medical advice	Justifies need for technological decision-support tools
Kim et al. (2021) [23]	Sports pharmacy systems	Clinical pharmacy in sport	Narrative review	Pharmacist service models	Emphasized pharmacist-led medication safety and anti-doping education	Connects healthcare technology with athlete protection
Alaranta et al. (2008) [18]	Prescription monitoring	Elite athletes	Narrative review	Prescription usage patterns	High prevalence of medication use with potential doping risk	Reinforces importance of digital screening systems
Yee et al. (2020) [22]	Digital pharmacy advisory systems	Athletes and pharmacists	Cross-sectional survey	Pharmacists	Pharmacists serve as critical medication advisors	Supports technological support tools for medication guidance
Sagoe et al. (2016) [24]	Technology-supported prevention programs	Youth and competitive athletes	Intervention study	Anti-doping education program participants	Education and structured interventions reduce doping risk behaviors	Complements digital prevention technologies

Note- AI = Artificial Intelligence; OCR = Optical Character Recognition; CAS = Court of Arbitration for Sport; WADA = World Anti-Doping Agency.

## Data Availability

No new data were created or analyzed in this study. The original contributions presented in this study are included in the article. Data sharing is not applicable to this article.

## Abbreviations

The following abbreviations are used in this manuscript:

CAS	Court of Arbitration for Sport
OCR	Optical Character Recognition
WADA	World Anti-Doping Agency
UI	User Interface
AI-OCR	Artificial Intelligence-Based Optical Character Recognition

## References

[1] Dyer B. (2015). The controversy of sports technology: A systematic review. SpringerPlus.

[2] Lee S.Y., Park J.H., Yoon J., Lee J.Y. (2023). A Validation Study of a Deep Learning-Based Doping Drug Text Recognition System to Ensure Safe Drug Use among Athletes. Healthcare.

[3] Dudek S., Koziak W., Makieła M., Bętkowska A., Kornacka A., Dudek W., Szostak K., Tomaka R., Byra A. (2025). Revolutionizing Sports: The Role of Wearable Technology and AI in Training and Performance Analysis. Qual. Sport.

[4] Glebova E., Su Y., Desbordes M., Schut P.O. (2025). Editorial: Emerging digital technologies as a game changer in the sport industry. Front. Sports Act. Living.

[5] Miah A. (2006). Rethinking enhancement in sport. Ann. N. Y. Acad. Sci..

[6] Guinness J., Bhattacharya D., Chen J., Chen M., Loh A. (2020). An Observational Study of the Effect of Nike Vaporfly Shoes on Marathon Performance. arXiv.

[7] Arderiu A., de Fondeville R. (2022). Influence of advanced footwear technology on sub-2 hour marathon and other top running performances. J. Quant. Anal. Sports.

[8] Xu Y., Zhu C., Fang Y., Lu Z., Song Y., Hu C., Sun D., Gu Y. (2025). The effects of different carbon-fiber plate shapes in shoes on lower limb biomechanics following running-induced fatigue. Front. Bioeng. Biotechnol..

[9] Dyer B. (2020). A Pragmatic Approach to Resolving Technological Unfairness: The Case of Nike’s Vaporfly and Alphafly Running Footwear. Sports Med. Open.

[10] Beck O.N., Taboga P., Grabowski A.M. (2016). Characterizing the Mechanical Properties of Running-Specific Prostheses. PLoS ONE.

[11] Rahnama L., Soulis K., Geil M.D. (2024). A review of evidence on mechanical properties of running specific prostheses and their relationship with running performance. Front. Rehabil. Sci..

[12] Siddiqui M., Alnaser I., Alluhydan K. (2023). Assessment of a Carbon Fiber Prosthetic Running Blade for Enhanced Reliability. Eksploat. Niezawodn. Maint. Reliab..

[13] Migliaccio G.M., Padulo J., Russo L. (2024). The Impact of Wearable Technologies on Marginal Gains in Sports Performance: An Integrative Overview on Advances in Sports, Exercise, and Health. Appl. Sci..

[14] Seçkin A.Ç., Ateş B., Seçkin M. (2023). Review on Wearable Technology in Sports: Concepts, Challenges and Opportunities. Appl. Sci..

[15] Triviño J.L.P. (2011). Gene Doping and the Ethics of Sport: Between Enhancement and Posthumanism. Int. J. Sports Sci..

[16] Pavot D. (2022). A Gap or Lacuna in the World Anti-Doping Code? Remarks on the CAS Interpretation in IOC, WADA, and ISU v. RUSADA, Kamila Valieva and Russian Olympic Committee (CAS OG 22-08, CAS OG 22-09, and CAS OG 22-10). Front. Sports Act. Living.

[17] Pitassi C., de Lacerda L.R. (2019). Technological capability of doping control laboratories: A metric proposal. Int. J. Sport. Policy Politics.

[18] Alaranta A., Alaranta H., Helenius I. (2008). Use of prescription drugs in athletes. Sports Med..

[19] Kim T., Kim Y.H. (2017). Korean national athletes’ knowledge, practices, and attitudes of doping: A cross-sectional study. Subst. Abus. Treat. Prev. Policy.

[20] Backhouse S.H., McKenna J. (2011). Doping in sport: A review of medical practitioners’ knowledge, attitudes and beliefs. Int. J. Drug Policy.

[21] Overbye M. (2016). Doping control in sport: An investigation of how elite athletes perceive and trust the functioning of the doping testing system in their sport. Sport Manag. Rev..

[22] Yee K.C., De Marco M., Salahudeen M.S., Peterson G.M., Thomas J., Naunton M., Kosari S. (2020). Pharmacists as a Source of Advice on Medication Use for Athletes. Pharmacy.

[23] Kim S., Cho S., Choi J., Lee Y.H., Rhie S. (2021). Sports Pharmacy: New Specialty of Pharmacists and Pharmaceutical Care Services. Korean J. Clin. Pharm..

[24] Sagoe D., Holden G., Rise E.N.K., Torgersen T., Paulsen G., Krosshaug T., Lauritzen F., Pallesen S. (2016). Doping prevention through anti-doping education and practical strength training: The Hercules program. Perform. Enhanc. Health.

[25] Baron D.A., Martin D.M., Abol Magd S. (2007). Doping in sports and its spread to at-risk populations: An international review. World Psychiatry.

[26] Park J.H., Yoon S.H., Yoon J.W., Lee S.Y., Lee H.G., Lee J.Y. (2022). Developement of Doping Drug Recognition System: Application of Deep Learning-Based OCR Technology. Korean J. Physic. Educ..

[27] Campbell M., McKenzie J.E., Sowden A., Katikireddi S.V., Brennan S.E., Ellis S., Hartmann-Boyce J., Ryan R., Shepperd S., Thomas J. (2020). Synthesis without meta-analysis (SWiM) in systematic reviews: Reporting guideline. BMJ.

[28] Siddaway A.P., Wood A.M., Hedges L.V. (2019). How to Do a Systematic Review: A Best Practice Guide for Conducting and Reporting Narrative Reviews, Meta-Analyses, and Meta-Syntheses. Annu. Rev. Psychol..

[29] Page M.J., McKenzie J.E., Bossuyt P.M., Boutron I., Hoffmann T.C., Mulrow C.D., Shamseer L., Tetzlaff J.M., Akl E.A., Brennan S.E. (2021). The PRISMA 2020 statement: An updated guideline for reporting systematic reviews. BMJ.

[30] Wohlin C., Kalinowski M., Romero Felizardo K., Mendes E. (2022). Successful combination of database search and snowballing for identification of primary studies in systematic literature studies. Inf. Softw. Technol..

[31] Rathbone J., Carter M., Hoffmann T., Glasziou P. (2015). Better duplicate detection for systematic reviewers: Evaluation of Systematic Review Assistant-Deduplication Module. Syst. Rev..

[32] Ouzzani M., Hammady H., Fedorowicz Z., Elmagarmid A. (2016). Rayyan-a web and mobile app for systematic reviews. Syst. Rev..

[33] Listiani D., Umar F., Riyadi S. (2024). Athletes’ (Anti) Doping Knowledge: A Systematic Review. Retos.

[34] Nicholls A.R., Lazuras L., Petrou M., Corazza O., Santos C., Nunes A.J., Rynkowski M., Martins J.F., Zandonai T., Kühn U. (2025). A Systematic Review on the Effectiveness of Anti-Doping Education for University Students. Emerg. Trends Drugs Addict. Health.

[35] Reynoso-Sánchez L.F., Molgado-Sifuentes A., Muñoz-Helú H., López-Walle J.M., Soto-García D. (2025). Effective Intervention Features of a Doping Prevention Program for Athletes: A Systematic Review with Meta-Analysis. Sports.

[36] Rico-González M., Pino-Ortega J., Clemente F.M., Arcos A.L. (2022). Guidelines for performing systematic reviews in sports science. Biol. Sport.

[37] Madsen D.Ø., Glebova E. (2025). Sports Industry 5.0: Reimagining sport through technology, humanity, and sustainability. Front. Sports Act. Living.

